# Schwannomatosis of the Spinal Accessory Nerve: A Case Report

**DOI:** 10.1055/s-0039-1685457

**Published:** 2019-04-26

**Authors:** Ramin A. Morshed, Anthony T. Lee, Young M. Lee, Cynthia T. Chin, Line Jacques

**Affiliations:** 1 Department of Neurological Surgery, University of California, San Francisco, California, United States; 2 Department of Neuroradiology, University of California, San Francisco, California, United States

**Keywords:** spinal accessory nerve tumor, schwannomatosis, schwannoma, neck mass, diffusion tensor imaging

## Abstract

Schwannomatosis is a distinct syndrome characterized by multiple peripheral nerve schwannomas that can be sporadic or familial in nature. Cases affecting the lower cranial nerves are infrequent. Here, the authors present a rare case of schwannomatosis affecting the left spinal accessory nerve. Upon genetic screening, an in-frame insertion at codon p.R177 of the Sox 10 gene was observed. There were no identifiable alterations in NF1, NF2, LZTR1, and SMARCB1. This case demonstrates a rare clinical presentation of schwannomatosis in addition to a genetic aberration that has not been previously reported in this disease context.

## Introduction

Schwannomas are benign peripheral nerve tumors that can be sporadic or familial in nature and typically associated with neurofibromatosis type-2 (NF2). 1 These tumors can affect any point along the peripheral nerve, including the cranial nerves, spinal roots, nerve plexi, and major peripheral nerves. They can cause symptoms such as pain, weakness, changes in sensation, and cranial nerve deficits, depending on the nerve in question.

Certain syndromes such as NF2 are associated with an increased frequency of schwannomas. For example, NF2 is associated with bilateral vestibular schwannomas. Schwannomatosis is another syndrome that appears to be a distinct entity from NF2 in that patients may have multiple schwannomas without any evidence of a vestibular nerve schwannoma or other findings associated with NF2. 2 3 4 Prior reports have demonstrated cases of schwannomatosis involving the cranial nerves, spinal roots, major peripheral nerves, and brachial or lumbar plexi. 3 5 However, as with neurofibromas, cases of schwannomatosis involving the lower cranial nerves (apart from the vestibulocochlear nerve) are rare. 3 6 7 8 9 Here, we report on a rare case of schwannomatosis involving the left spinal accessory nerve and provide imaging findings and a description of the surgical approach.

## Case Report

### History, Physical Examination, and Imaging Findings

**Fig. 1:**
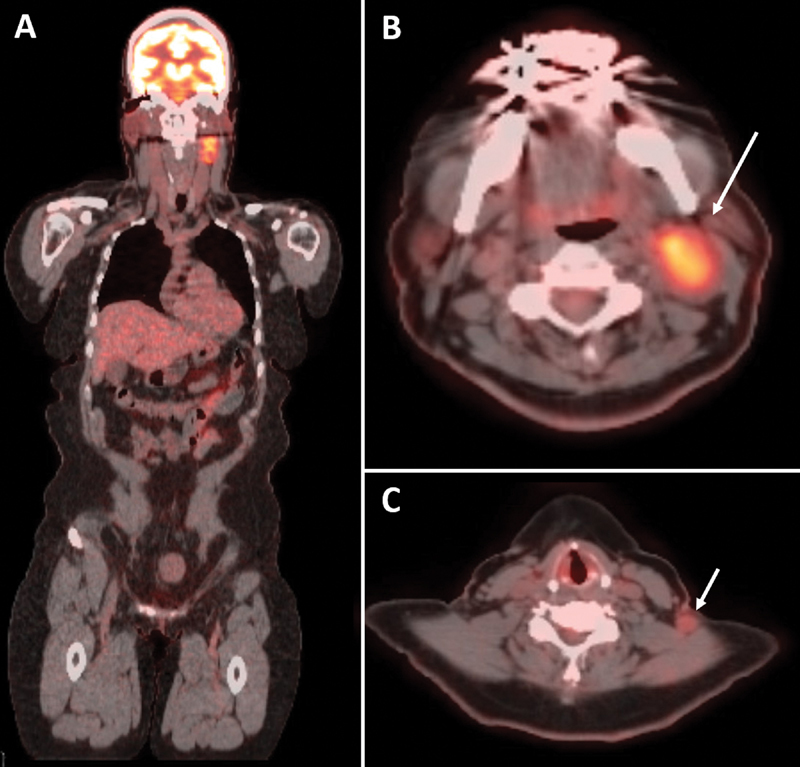
( **A–C** ) Positron emission tomography–computed tomography (PET CT) was performed, which demonstrated increased fluoro-2-deoxy-d-glucose uptake in both masses (standardized uptake value of 7.3 for the larger mass and 2.7 for the smaller mass).

**Fig. 2:**
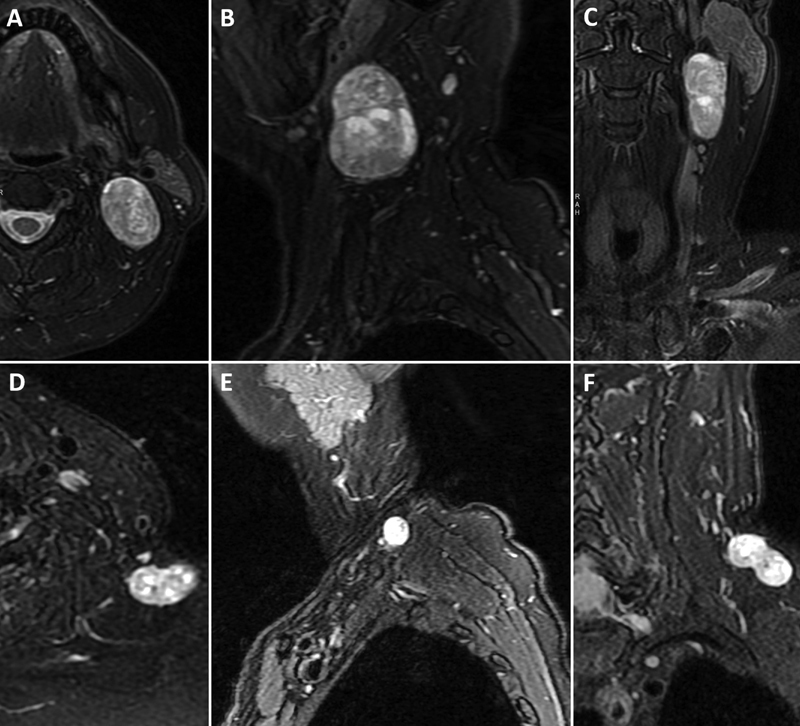
Magnetic resonance imaging (MRI) neurogram demonstrated a larger 4.6 × 3.2 × 2.5 cm mass deep to the left sternocleidomastoid muscle just below the angle of the mandible **(A–C)** and a bilobed 2.4 × 2.2 × 1.3 cm mass in the left posterior supraclavicular region **(D–F)** .

**Fig. 3:**
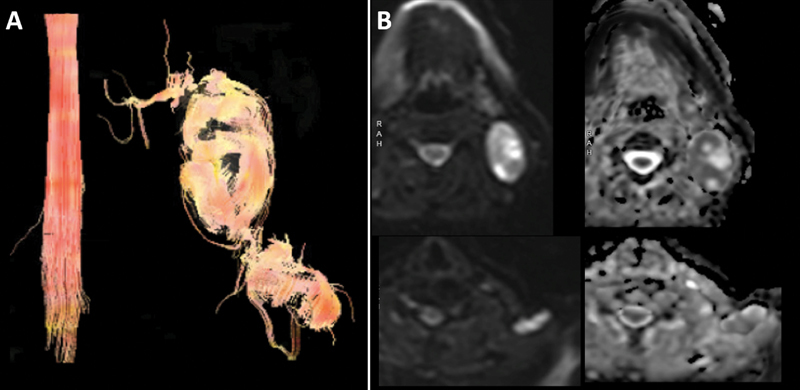
Further imaging characterization of lesions. **(A)** Diffusion tensor imaging with tractography demonstrated abnormal thickened nerve fibers coursing through the two spinal accessory nerve tumors. **(B)** Axial diffusion weight imaging (left) and apparent diffusion coefficient (ADC) images (right) of the lesions. ADC values were 1.3 × 10 ^−6^ and 1.8 × 10 ^−6^ mm ^2^ /second for the larger and smaller masses, respectively.

This case is of a 55-year-old female who initially presented with a palpable left neck mass. The mass had been noted by the patient 8 years ago and had progressively grown in size. A computed tomography (CT) of the neck was obtained, which demonstrated a 3.5 × 2.3 × 4.6 cm lesion deep to the left sternocleidomastoid (SCM) muscle in addition to a smaller 1 × 1 × 1.8 cm left posterior neck-enhancing mass. A fine needle aspiration (FNA) had been performed 2 years prior at an outside hospital with pathology indicating a low-grade spindle cell proliferation. She was thus referred to our institution for further evaluation. At the time of initial presentation to our group, the patient had noted dysphagia, left ear ache and tinnitus, and neck pain localized to the two masses. A positron emission tomography (PET) CT was performed, which demonstrated increased fluoro-2-deoxy- d -glucose uptake in the high cervical mass and, to a lesser degree, in the mass located within the posterior triangle of the neck ( Fig. 1 ). The outside tissue blocks were reviewed at our institution and felt to be consistent with a peripheral nerve sheath tumor compatible with schwannoma from both lesions. Because of the patient's ongoing symptoms and because malignancy could not be completely excluded based on the results of the prior FNA, the decision was made to proceed with surgical excision of both lesions. Prior to proceeding, a magnetic resonance neurogram was obtained ( Fig. 2 ). Tractography demonstrated that the two lesions appeared to originate from the spinal accessory nerve and that apparent diffusion coefficient values were elevated in both masses, supporting the diagnosis of a less aggressive tumor ( Fig. 3 ).

### Treatment Course

**Fig. 4:**
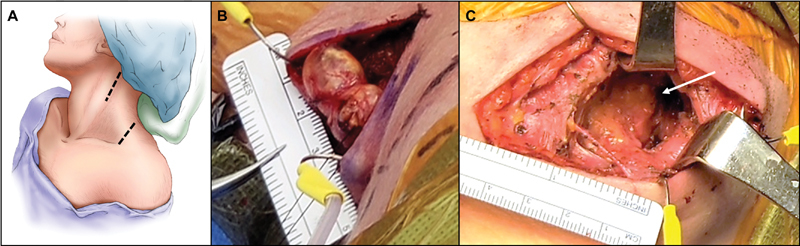
Intraoperative findings. **(A)** Two separate incisions were required to remove both lesions. **(B)** The smaller bilobed lesion mass was accessed through the posterior triangle of the neck and was located on the distal spinal accessory nerve. **(C)** The larger more proximal mass was approached medial to the sternocleidomastoid muscle in the upper neck. Both masses underwent a gross total resection.

The patient was taken to the operating room for surgical resection. The patient was positioned with her head turned slightly to the right with the neck extended ( Fig. 4A ). The smaller lesion was approached first through the posterior triangle of the neck. Stimulation mapping of the tumor was conducted. The tumor was found to be located on the distal spinal accessory (cranial nerve XI) nerve, with evidence of trapezius activation with nerve stimulation ( Fig. 4B ). After identifying no overlying nerve fibers, the tumor was removed en bloc. A separate incision was made in the upper cervical region to approach the larger second mass located lateral and deep to the SCM muscle, which was reflected medially ( Fig. 4C ). During dissection of the tumor away from the nerve, motor evoked potentials (MEPs) to the trapezius were lost. As the tumor was of significant size, view of the proximal aspect of the afferent nerve was initially obstructed. Distally, the tumor was mapped, and the fascicle of origin was identified, which appeared to activate the SCM. After significant debulking of the mass, the proximal fascicle of origin was identified but did not provide any muscle activation after stimulation. The tumor was therefore removed in its entirety. All parameters for brachial plexus monitoring remained stable.

### Pathological Findings and Clinical Outcome

Pathology for both lesions was consistent with schwannoma without malignant features. Next-generation sequencing analyzing the coding regions of 479 cancer genes as well as select introns of 47 genes using the UCSF 500 Cancer Gene Test revealed a small in-frame insertion at codon p.R177 of the Sox 10 gene. There were no identifiable alterations in NF1, NF2, LZTR1, SMARCB1, and TRAF7 genes. Despite the change in MEPs, the patient was noted to be full strength in all muscle groups in the left upper extremity including shoulder shrug and head turning immediately postoperatively. At follow-up, her neck pain and prior dysphagia had improved significantly.

## Discussion

Schwannomatosis is a syndrome characterized by multiple peripheral nerve schwannomas usually without involvement of the vestibular nerve and can be sporadic or familial in nature. In a retrospective analysis of 87 patients with schwannomatosis, 89% had peripheral tumors, 74% had spinal tumors, and 9% had intracranial nonvestibular tumors. 9 The typical age of presentation is between 30 and 60 years, with pain being the most common presenting symptom. 9 Prevalence has been reported to be approximately 1 in 140,000 to 150,000, 3 10 and life expectancy is reported to be near-normal (76.9 years) and significantly longer than for patients with NF2. 3

Although there is phenotypic overlap with the other syndromes under neurofibromatosis, such as NF2, schwannomatosis is a distinct entity. Diagnostic criteria include at least two nonintradermal anatomically distinct schwannomas (at least one histologically confirmed) with no radiographic evidence of bilateral vestibular schwannomas on MRI and NF2 mutation negative in a patient at least 30 years of age. Other criteria include one biopsy-proven nondermal schwannoma or intracranial meningioma plus a first-degree relative with schwannomatosis. Although diagnostic criteria initially excluded patients with vestibular schwannomas, recent reports suggest that schwannomatosis patients may still develop unilateral vestibular schwannomas. 11 12 Furthermore, intracranial meningiomas or cutaneous neurofibromas do not exclude a diagnosis of schwannomatosis, although other features of other neurofibromatosis syndromes such as Lisch's nodules and café au lait macules are not present.

The most well-described genetic alteration is a mutation in the SMARCB1 gene or LZTR1 gene on chromosome 22q11.2. 13 In our case, these mutations were not present and instead a mutation in SOX10 was observed. While SOX10 has been used previously as an identifying marker for schwannomas, mutation of SOX10 as a contributor to the pathogenesis of schwannomatosis, to our knowledge, has not been previously reported.

Management of schwannomatosis patients represents a therapeutic challenge. Typically, surgical intervention is indicated for symptomatic lesions. 5 We recommend the use of intraoperative neuromonitoring for all cases with the use of stimulation to help identify functional fascicles that should be preserved. In this case, although injury was sustained to the fibers of the spinal accessory nerve, trapezius muscle innervation was maintained likely because of the additional innervation of the trapezius muscle by branches of the cervical plexus. 14 Asymptomatic tumors may be observed, but close imaging surveillance is required. Further management considerations include referral to a pain management specialist. In the study by Merker et al, 68% of schwannomatosis experienced chronic pain. Despite surgery and pain medications, the majority did not become pain-free. 9 Thus, ongoing treatment under the care of a pain management specialist is important from a quality-of-life standpoint. Additionally, higher rates of depression and anxiety can be seen in these patients, likely in the setting of chronic pain, the source of which is often undiagnosed for years. Active surveillance and treatment of mood disorders is therefore another critical component of medical care. 9

## Conclusion

Here, we report a rare case of schwannomatosis in addition to a genetic aberration that has not been previously reported in this disease context. Using intraoperative mapping and microsurgical technique, safe resection may be attempted for symptomatic lesions.
